# How Do Glycine‐Induced Bent Structures Influence Hierarchical Nanostructuring and Suprastructural Handedness in Short Peptide Assembly?

**DOI:** 10.1002/advs.202413602

**Published:** 2025-02-25

**Authors:** Xinfeng Ju, Kai Qi, Yan Wang, Limin Zhang, Yingyu Wang, Muhan Wang, Jiqian Wang, Jun Zhang, Jian R. Lu, Hai Xu, Yurong Zhao

**Affiliations:** ^1^ Department of Biological and Energy Chemical Engineering China University of Petroleum (East China) 66 Changjiang West Road Qingdao 266580 China; ^2^ Biological Physics Group Department of Physics and Astronomy The University of Manchester Manchester M13 9PL UK; ^3^ Department of Civil Engineering Qingdao University of Technology Qingdao 266033 China; ^4^ School of Material Science and Engineering China University of Petroleum (East China) 66 Changjiang West Road Qingdao 266580 China

**Keywords:** achiral glycine, bent structures, hierarchical nanostructuring, peptide self‐assembly, supramolecular handedness

## Abstract

Despite the multiple roles of flexible and achiral Gly in regulating protein architectures and functions, its high flexibility is seldom exploited as a structural modulator in the design of self‐assembling peptides. By using minimalistic peptide sequences, the effects of Gly insertions are investigated on the molecular conformation and the supramolecular morphology, focusing on Gly‐induced bent structures and their impact on self‐assembled nanostructures and handedness. Different backbone bending degrees are generated by varying Gly position, which in turn resulted in distinct hydrogen bonding modes and residue shifting upon dimerization, eventually leading to *β*‐sheets and nanofibrils with opposite handedness. The bent structures are revealed to be primarily caused by van der Waals interactions between either the side chains themselves or the side chain and the local backbone around the inserted Gly, in sharp contrast to canonical *β*‐turns stabilized by intrastrand hydrogen bonding. Hence, changing the side chain orientations of adjacent residues by chiral substitution can destabilize the bent structures, leading to wide ribbons with suprastructural chiral racemization. This study not only helps understand the versatile roles of Gly in protein architectures but also serves as a paradigm for tuning peptide supramolecular nanostructures and handedness via Gly insertion.

## Graphical abstract

How does flexible and achiral glycine affect suprastructural handedness in peptide self‐assembly? A series of short peptides containing glycine is designed, and different molecular conformations caused by varying the position of glycine can lead to different hydrogen bonding patterns upon forming *β*‐sheets, eventually resulting in variations in their supramolecular morphology and chirality.

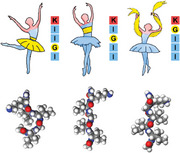

## 1 Introduction

Glycine (Gly or G) is the only one achiral amino acid among the 20 common ones. Because of its side group being hydrogen (H), Gly shows a high degree of freedom in protein folding and can provide greater flexibility than all other natural amino acid residues within a sequence. As a result, Gly is frequently encountered in many local structures of globular proteins, including turns, loops, and bends. For example, the third residue (*i* + 2) in the Type II turn is dominated by Gly while the type I turn exhibits a strong preference for Gly at *i* + 3.^[^ ^1^ ^]^ Evolution facilitates the use of Gly‐rich loops for nucleotide binding in proteins.^[^ ^2^ ^]^ These Gly‐rich structures play multiple roles in protein architectures and biological functions, such as changing polypeptide chain direction and conferring a protein globularity rather than linearity,^[^ ^1^, ^3^ ^]^ connecting successive regular secondary structures to form compact super‐secondary structure motifs within protein (e.g., *βαβ*, *β* meanders and barrels, and helix‐turn‐helix),^[^ ^4^, ^5^ ^]^ and providing the proper degree of conformational flexibility and dynamics for interacting with ligands and other biomolecules.^[^ ^2^, ^6^ ^]^ Additionally, Gly is particularly rich in fibrous structural proteins such as collagen (Gly‐Xaa‐Yaa repeats) and silk fibroin (Gly‐Xaa repeats) with superior mechanical properties, in which the conformation of Gly tends to be extended.^[^ ^7^ ^]^ The extended conformations of Gly and its very small side chain not only favor the close packing of the collagen triple helix or the tight stacking of the fibroin *β*‐sheets but also enable optimal inter‐strand or inter‐sheet hydrogen bond formation at higher structural scales (beyond molecules).^[^ ^7^, ^8^ ^]^

Given its extraordinary versatility in protein architectures and functions, Gly is also expected to play an important role in peptide assembly, especially in tuning self‐assembled peptide architectures and material properties. Relative to other amino acids with strong propensity for *α*‐helix or *β*‐sheet (e.g., alanine (Ala or A), leucine (Leu or L), isoleucine (Ile or I), and phenylalanine (Phe or F)) or with specific physiochemical and biochemical properties (e.g., arginine (Arg or R), histidine (His or H), lysine (Lys or K), glutamic acid (Glu or E), aspartic acid (Asp or D), and tyrosine (Tyr or Y)), however, this residue has been seldom considered as the key moiety in the design of short self‐assembling peptides. One exception is the G_n_D_2_ peptides (n = 4, 6, 8, and 10, D denotes aspartic acid) designed by Santoso et al., which self‐assembled into nanotubes and nanovesicles.^[^ ^9^ ^]^ For these molecules, consecutive Gly residues were simply assumed to constitute the hydrophobic tails and play the same role as the six alanine and valine residues in surfactant‐like peptides such as A_6_K and V_6_D.^[^ ^9^, ^10^ ^]^ There is a lack of assessment of the effect of the Gly specificity or flexibility on the whole molecular conformation for these short peptides.

At the same time, even for a very short self‐assembling peptide sequence, little is known about the effect of Gly insertion on its supramolecular nanostructures and morphology. We have recently explored the feasibility of this investigation by inserting Gly at the interface of the hydrophobic and hydrophilic moieties of I_3_K.^[^ ^11^ ^]^ We found that this simple Gly insertion could destabilize the effect of the C‐terminal Lys chirality (chiral truncation), by inducing distinct molecular conformations. Such an unusual outcome prompts us to conjecture what will happen when varying the Gly position within the sequence, in particular, when the residue being buried in the hydrophobic and structurally directed I_3_ segment.

**Scheme 1:**
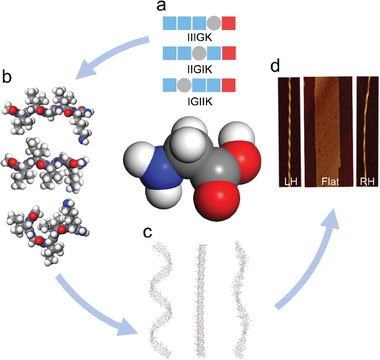
Schematic illustrations showing how Gly insertions within the I_3_K sequence (a) cause the formation of diverse molecular conformations represented by bent and extended structures (b), distinct *β*‐sheet assemblies (c), and supramolecular nanostructures and handedness (d). LH and RH denote left‐ and right‐handed fibrils, respectively, and Flat denotes flat ribbon.

To gain a general prospective of the impact of Gly insertion, we have designed a series of short amphiphilic peptides by moving the Gly position within the I_3_K sequence, including *^L^* IG*^L^* I_2_ *^L^* K, *^Da^* IG*^L^* I_2_ *^L^* K, *^L^* IG*^L^* I_2_ *^D^* K, *^Da^* IG*^L^* I_2_ *^D^* K, *^L^* I_2_G*^L^* I*^L^* K, *^L^* I_2_G*^L^* I*^D^* K, *^L^* I_3_G*^L^* K, and *^L^* I_3_G*^D^* K (*^L^* I and *^Da^* I denote L‐form and D‐allo‐form isoleucine, respectively, and *^L^* K and *^D^* K denote L‐form and D‐form lysine, respectively). Through the combined use of microscopy imaging and theoretical simulations, we investigated the hierarchical self‐assembly processes of these Gly‐containing pentapeptides and found that Gly insertions at different positions caused the formation of diverse molecular conformations (extended and bent structures), which in turn influenced subsequent dimerization and *β*‐sheet assembly, eventually resulting in distinct nanostructures and suprastructural handedness (**Scheme** **1**). Despite the short lengths of these designed peptides compared to large proteins and long polypeptides, they serve as minimalist sequences, and their self‐assembly can provide new insights that are useful in understanding the versatile roles of Gly in protein architectures.^[^ ^12^ ^]^ The incorporation of D‐form amino acids can help clarify how the side chain orientations affect the backbone bending and supramolecular handedness.

## 2 Results and Discussion

### 2.1 Self‐Assembled Morphologies and Suprastructural Handedness

**Figure 1:**
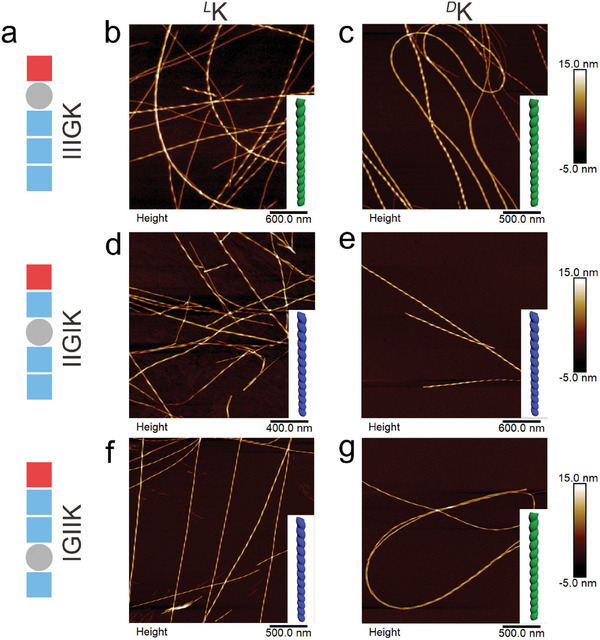
Suprastructural handedness of peptide assemblies revealed by AFM height imaging. a) Peptide sequences and schematic illustrations of their molecular shapes. b,c) right‐handed *^L^* I_3_G*^L^* K and *^L^* I_3_G*^D^* K nanofibrils, respectively. d,e) left‐handed *^L^* I_2_G*^L^* I*^L^* K and *^L^* I_2_G*^L^* I*^D^* K nanofibrils, respectively. f,g) left‐handed *^L^* IG*^L^* I_2_ *^L^* K and right‐handed *^L^* IG*^L^* I_2_ *^D^* K nanofibrils, respectively. The peptides were dissolved in water at the concentration of 8 mm, with the solution pH of 7.0. Note that decreasing or increasing the peptide concentration to 4 or 16 mm did not change the fibril morphology and handedness. The peptide solutions were incubated for 1 week at ambient conditions prior to AFM measurements.

To determine the nanostructures self‐assembled from these constitution‐ and stereo‐isomeric pentapeptides and their suprastructural handedness, we first performed atomic force microscopy (AFM) and transmission electron microscopy (TEM) characterizations. The six peptides, *^L^* IG*^L^* I_2_ *^L^* K, *^L^* IG*^L^* I_2_ *^D^* K, *^L^* I_2_G*^L^* I*^L^* K, *^L^* I_2_G*^L^* I*^D^* K, *^L^* I_3_G*^L^* K, and *^L^* I_3_G*^D^* K, readily formed thin nanofibrils with diameters of ≈10 nm (**Figure** **1**; Figure S1, Supporting Information). However, their nanofibrils showed distinct helical handedness. For *^L^* I_3_G*^L^* K and *^L^* I_3_G*^D^* K, right‐handed nanofibrils were the dominant structures (Figure 1b,c), with left‐handed and flat nanofibrils being occasionally observed (<10% in population, Figure S2, Supporting Information), consistent with our previous observations.^[^ ^11a^ ^]^ In contrast, the nanofibrils formed by *^L^* I_2_G*^L^* I*^L^* K and *^L^* I_2_G*^L^* I*^D^* K were exclusively left‐handed (Figure 1d,e). Moreover, when Gly was moved to the second position (Gly2), *^L^* IG*^L^* I_2_ *^L^* K formed left‐handed nanofibrils while *^L^* IG*^L^* I_2_ *^D^* K self‐assembled into right‐handed ones (Figure 1f,g). Given the high flexibility of Gly, these dramatic changes in fibril handedness upon varying the Gly position within the sequence suggested the different molecular conformations and chirality adopted by these isomers, which may in turn influence their subsequent assembly and suprastructural handedness.

### 2.2 Molecular conformations, Gly‐Induced Backbone Bending, and Interactions Implicated

**Figure 2:**
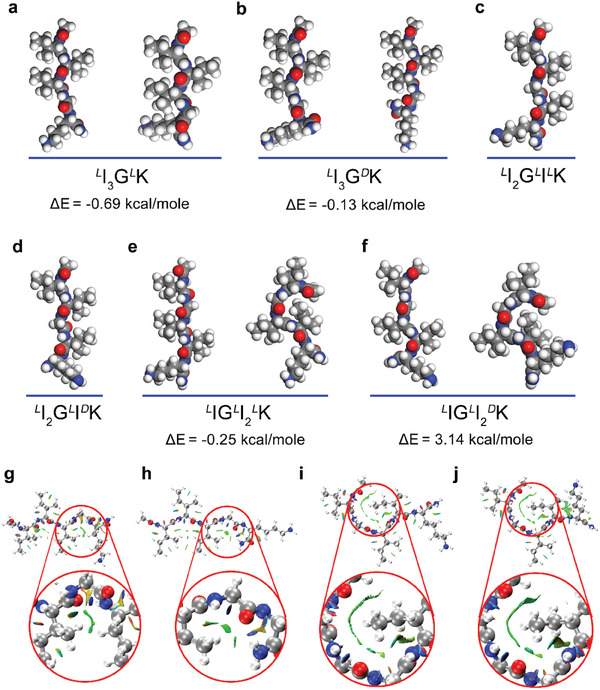
Stable conformations and visual analysis of intramolecular non‐covalent interactions of single peptides. a‐f) Stable conformations and the energy gaps between the extended and bent conformations for a) *^L^* I_3_G*^L^* K, b) *^L^* I_3_G*^D^* K, e) *^L^* IG*^L^* I_2_ *^L^* K, and f) *^L^* IG*^L^* I_2_ *^D^* K. g‐j) The RDG isosurfaces for the bent chains of g) *^L^* I_3_G*^L^* K, h) *^L^* I_3_G*^D^* K, i) *^L^* IG*^L^* I_2_ *^L^* K, and j) *^L^* IG*^L^* I_2_ *^D^* K, respectively. Three residues in *^L^* I_3_G*^L^* K and *^L^* I_3_G*^D^* K while four residues in *^L^* IG*^L^* I_2_ *^L^* K and *^L^* IG*^L^* I_2_ *^D^* K were involved in their bent structures. The RDG isosurfaces for the extended chains are given in Figure S3 (Supporting Information). Atoms coloring scheme for molecular structures is: red, oxygen; blue, nitrogen; white, hydrogen, and gray, carbon. Red, green, and blue patches in the RDG isosurfaces represent repulsive steric hindrance, vdW interaction, and attractive H‐bonding, respectively.

To identify the molecular explanation for the chirality complexity associated with these peptide fibrils, we then conducted quantum chemistry (QC) calculations, in combination with molecular dynamic (MD) simulations. *^L^* I_3_G*^L^* K, *^L^* I_3_G*^D^* K, *^L^* IG*^L^* I_2_ *^L^* K, and *^L^* IG*^L^* I_2_ *^D^* K displayed two stable molecular conformations, corresponding to extended and bent chains, respectively (**Figure** **2**a,b,e,f). The extended conformations of single *^L^* I_3_G*^L^* K, *^L^* I_3_G*^D^* K, and *^L^* IG*^L^* I_2_ *^L^* K molecules (Figure 2a,b,e, left) were slightly more stable than their bent conformations (Figure 2a,b,e, right). In contrast, the extended conformation of the *^L^* IG*^L^* I_2_ *^D^* K monomer (Figure 2f, left) was less stable than its bent conformation (Figure 2f, right), with a relatively large energy gap of 3.14 kcal mol^−1^ between them. When Gly was located in the middle position (Gly3) within the sequence, however, only one stable conformation, i.e., an extended chain, was observed for *^L^* I_2_G*^L^* I*^L^* K or *^L^* I_2_G*^L^* I*^D^* K, respectively (Figure 2c,d).

To find out the rationale underpinning the formation of the bent structures, the reduced density gradient (RDG) method, developed by Yang et al.,^[^ ^13^ ^]^ was applied to analyze the intramolecular non‐covalent interactions involved (Figure 2g–j). As expected, chain bending occurred around the Gly residue due to its lack of bulky side chains, clearly driven by van der Waals (vdW) interactions between either the side chains of its preceding and following residues (Ile3 and Lys5) for *^L^* I_3_G*^L^* K and *^L^* I_3_G*^D^* K or the Ile4 side chain and the N‐terminal backbone (Ile1, Gly2, and Ile3) for *^L^* IG*^L^* I_2_ *^L^* K and *^L^* IG*^L^* I_2_ *^D^* K (Figure 2g–j, green patches in the enlarged regions). However, the main chain CO (*i*) to NH (*i* + 3) hydrogen bonding, which is often present in *β*‐turns and *β*‐bends of proteins,^[^ ^1^, ^3^ ^]^ was not observed in these bent structures, suggesting a radical difference between them.

**Figure 3:**
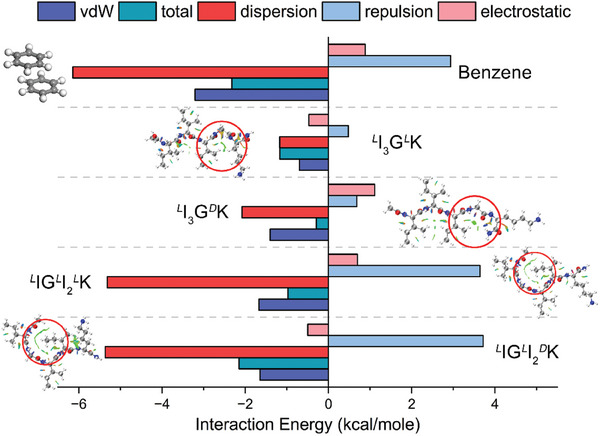
EDA‐FF analysis of the local bent regions within single *^L^* I_3_G*^L^* K, *^L^* I_3_G*^D^* K, *^L^* IG*^L^* I_2_ *^L^* K, and *^L^* IG*^L^* I_2_ *^D^* K molecules, as indicated by the red circles. The benzene dimer with a parallel‐displaced configuration acted as a reference. Such an analysis resulted in the generation of electrostatic energy, exchange repulsion energy, and dispersion energy. The sum of the latter two energies represents the vdW interaction energy. Atoms coloring scheme for molecular structures is: red, oxygen; blue, nitrogen; white, hydrogen, and gray, carbon.

To further assess the nature as well as the magnitude of the weak interactions involved in the bent structures, we performed energy decomposition analysis based on the molecular forcefield (EDA‐FF, please see Supporting Information for details).^[^ ^14^ ^]^ The weak interaction energy of the bent regions (denoted by red circles in Figures 2g–j and **3**) can be decomposed into three items, i.e., electrostatic energy, exchange repulsion energy, and dispersion energy. Among them, the sum of exchange repulsion energy and dispersion energy is equal to the vdW interaction energy. Both total interaction energies and vdW interaction energies from the four bent structures were in negative values (**Figure** 3), suggesting the conformations are thermodynamically favorable. The electrostatic energies were small from these bent structures and contributed little to their total energies, consistent with the lack of hydrogen bonding as observed above. Importantly, the dispersion and repulsion energies of the *^L^* IG*^L^* I_2_ *^L^* K and *^L^* IG*^L^* I_2_ *^D^* K bends were comparable to those of the benzene dimer with a parallel‐displaced configuration. As the simplest π‐π stacking system, the benzene dimer is stabilized by strong vdW interactions including the exchange repulsion and dispersion attractive interactions.^[^ ^15^ ^]^ In particular, the total energy of the bent conformation of *^L^* IG*^L^* I_2_ *^D^* K was very close to that of the benzene dimer, consistent with a higher tendency for single *^L^* IG*^L^* I_2_ *^D^* K molecule to form the bent structure.

### 2.3 From Monomers to Dimers

**Figure 4:**
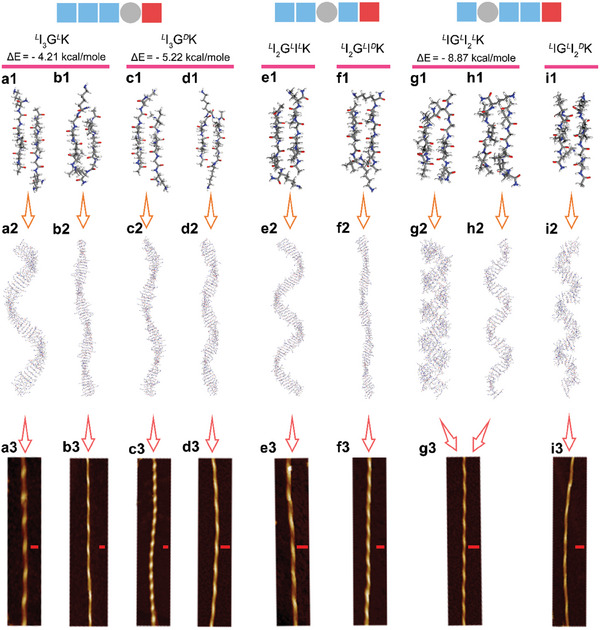
Stable peptide dimers, multi‐stranded *β*‐sheets, and self‐assembled fibrils. a1‐i1) Optimized dimers. The dimers in (a1,c1,e1,f1,g1) are extended dimers, those in (b1,d1) are bent dimers, and those in (h1,i1) are mixed dimers. The binding energy gaps between two dimers of *^L^* I_3_G*^L^* K, *^L^* I_3_G*^D^* K, and *^L^* IG*^L^* I_2_ *^L^* K are also given, respectively. a2–i2) Multi‐stranded *β*‐sheets evolved from the dimers shown in (a1–i1). a3–g3,i3) Fibrils and their suprastructural handedness determined from AFM measurements. Red scale bar: 50 nm.

Based on these stable conformations of monomers, we then moved to their dimerization, which were primarily driven by interstrand hydrogen bonding (H‐bonding). Given that antiparallel *β*‐sheets are generally more stable than parallel *β*‐sheets, together with the fact that antiparallel arrangements can greatly alleviate the electrostatic repulsion of charge residues of amphiphilic peptides upon dimerization, we have chosen the dimers with antiparallel H‐bonding mode for further simulations. For pentapeptides, there are two pairs of CO and NH groups on the one side of the strand and three pairs of CO and NH groups on the opposite side. As a result, there should produce a one‐residue registry shift between the two strands upon dimerization. Configurational search using the Molclus programme allows the determination of stable dimers with energy minima.^[^ ^16^ ^]^ For *^L^* I_3_G*^L^* K or *^L^* I_3_G*^D^* K, we observed two stable dimers, consisting of two extended chains and two bent chains, termed as extended and bent dimers, respectively (**Figure** **4**a1–d1). Consistent with their monomers, the extended dimer was more stable than the bent dimer, with the binding energy gap being −4.21 and −5.22 kcal mol^−1^ between them for *^L^* I_3_G*^L^* K and *^L^* I_3_G*^D^* K, respectively. For *^L^* I_2_G*^L^* I*^L^* K or *^L^* I_2_G*^L^* I*^D^* K, only one stable extended dimer was obtained (Figure 4e1,f1), the same as its monomer. Importantly, it was the Lys5 residues that shifted outward in the above six dimers.

Despite two stable dimers obtained for *^L^* IG*^L^* I_2_ *^L^* K, their detailed structures differed substantially from those of *^L^* I_3_G*^L^* K and *^L^* I_3_G*^D^* K, i.e., a mixed dimer consisting of one extended chain and one bent chain (Figure 4g1) and an extended dimer (Figure 4h1). Second, the mixed dimer was more stable than the extended dimer, with a binding energy gap of −8.87 kcal mol^−1^. Third, although it was still the Lys5 residues that shifted outward in the extended dimer, the Ile1 residues shifted outward in the mixed dimer. It was interesting that for *^L^* IG*^L^* I_2_ *^D^* K, only one stable mixed dimer was obtained in our configurational search, also with the Ile1 residues shifting outward within this mixed dimer (Figure 4i1).

**Figure 5:**
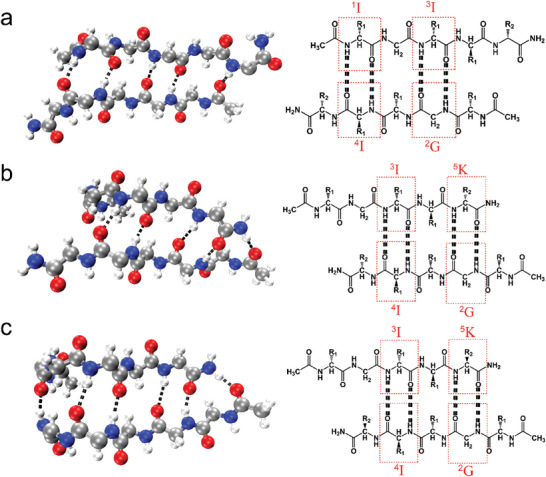
One‐residue registry shift between strand upon dimerization. a) Lys5 shifting out and interstrand H‐bonding within the *^L^* IG*^L^* I_2_ *^L^* K extended dimer. b) Ile1 shifting out and interstrand H‐bonding within the *^L^* IG*^L^* I_2_ *^L^* K mixed dimer. c) Ile1 shifting out and interstrand H‐bonding within the *^L^* IG*^L^* I_2_ *^D^* K mixed dimer. For the dimer structures shown on the left, the side chains are omitted to clearly show the backbone orientation and interstrand H‐bonds. Atoms coloring scheme for these structures is: red, oxygen; blue, nitrogen; white, hydrogen, and gray, carbon, and the black dotted lines represent interstrand H‐bonds. The residues participating in interstrand H‐bonding are clearly explained with the right schematic illustrations (R_1_ and R_2_ denote the side chains of Ile and Lys, respectively). The one‐residue shift upon dimerization is given in Figure S4 (Supporting Information) for *^L^* I_3_G*^L^* K, *^L^* I_3_G*^D^* K, *^L^* I_2_G*^L^* I*^L^* K, and *^L^* I_2_G*^L^* I*^D^* K.

It was clear that for the above dimers consisting of two extended chains, Lys5 residues instead of Ile1 residues shifted outward upon packing through interstrand H‐bonding (**Figure** **5**a; Figure S4a,c,e,f, Supporting Information). This can be explained by the much stronger propensity for *β*‐sheet H‐bonding of *β*‐branched Ile as well its high hydrophobicity, compared to unbranched, hydrophilic Lys.^[^ ^17^ ^]^ For the *^L^* I_3_G*^L^* K or *^L^* I_3_G*^D^* K bent dimers consisting of two bent chains, their C‐terminal bending also disfavored Lys5 residues to participate in interstrand H‐bonding (Figure S4b,d, Supporting Information). For *^L^* IG*^L^* I_2_ *^L^* K, however, two opposite one‐residue registry shifts, i.e., the shifting out of Lys5 and Ile1 residues, happened for its extended and mixed dimers, respectively. As expected, H‐bonds were formed by the Ile4 and Gly2 residues of one strand with the Ile1 and Ile3 residues of the other strand for the extended dimer (Figure 5a). For the *^L^* IG*^L^* I_2_ *^L^* K mixed dimer, the N‐terminal bending of the bent strand disallowed its Ile1 residue to participate in interstrand H‐bonding, and instead, H‐bonds were formed by the Ile3 and Lys5 residues of the bent strand with the Ile4 and Gly2 residues of the extended strand (Figure 5b). The interstrand H‐bonding mode for the *^L^* IG*^L^* I_2_ *^D^* K mixed dimer was similar to that of the *^L^* IG*^L^* I_2_ *^L^* K mixed dimer (Figure 5c). Because the N‐terminal backbone was unavailable for interstrand H‐bonding due to its significant bending for *^L^* IG*^L^* I_2_ *^L^* K and *^L^* IG*^L^* I_2_ *^D^* K, it was unlikely to form a stable dimer consisting of two bent chains, consistent with the above configurational search.

### 2.4 From Dimers to Multi‐Stranded *β*‐Sheets and Fibrils

With the stable dimers acting as the fundamental structural units, multi‐stranded *β*‐sheets were constructed along the interstrand H‐bonding direction using the Kabsch algorithm.^[^ ^18^ ^]^ After structural optimization, these *β*‐sheets displayed diverse helical twisting (Figure 4a2–i2). The sheets constructed from the extended and bent dimers of *^L^* I_3_G*^L^* K or *^L^* I_3_G*^D^* K showed right‐ and left‐handed spirals, respectively (Figure 4a2–d2), well consistent with nanofibril (*β*‐sheet laminates) handedness from experimental observations (Figure 4a3–d3). The *^L^* I_2_G*^L^* I*^L^* K and *^L^* I_2_G*^L^* I*^D^* K sheets comprised of their extended dimers exhibited left‐handed helicity (Figure 4e2,f2) and their nanofibrils showed left‐handedness (Figure 4e3,f3). Interestingly, the sheets constructed from the extended and mixed dimers of *^L^* IG*^L^* I_2_ *^L^* K all displayed left‐handedness (Figure 4g2,h2). Consistent with this theoretical analysis, only left‐handed helical nanofibrils were observed in experiments (Figure 4g3). For *^L^* IG*^L^* I_2_ *^D^* K, the sheet comprised of its mixed dimers exhibited a right‐handed spiral (Figure 4i2), also in good line with experimental observations (Figure 4i3).

Overall, the excellent agreement between our simulations and experimental observations validated the theoretical methodology adopted. Further analyses on the simulation results allowed us to identify the distinct roles of the chirality of the C‐terminal Lys5 in regulating handedness for the different amphiphilic peptides. For *β*‐sheets with the Lys5 residues shifting out, the residues at the C‐termini did not participate in interstrand H‐bonding and the twisting direction of the sheets was determined by the rest residues. Thus, *β*‐sheets and nanofibrils of both *^L^* I_2_G*^L^* I*^L^* K and *^L^* I_2_G*^L^* I*^D^* K exhibited left‐handedness, irrespective of the chirality of Lys5. Note that the coexistence of the right‐ and left‐handedness for *^L^* I_3_G*^L^* K or *^L^* I_3_G*^D^* K *β*‐sheets and nanofibrils was caused by the two states (extended and bent) of achiral Gly4 within the backbone rather than the chirality of Lys5.^[^ ^11a^ ^]^ For *β*‐sheets with the Ile1 residues shifting out due to the N‐terminal bending, the Lys5 residues at the C‐termini did participate in interstrand H‐bonding and the twisting of the sheets was thus dictated by their chirality. As a result, *β*‐sheets and nanofibrils of *^L^* IG*^L^* I_2_ *^L^* K and *^L^* IG*^L^* I_2_ *^D^* K exhibited left‐ and right‐handedness, respectively.

### 2.5 Loss of Bent Structures by Further Peptide Design and Suprastructural Chiral Racemization

**Figure 6:**
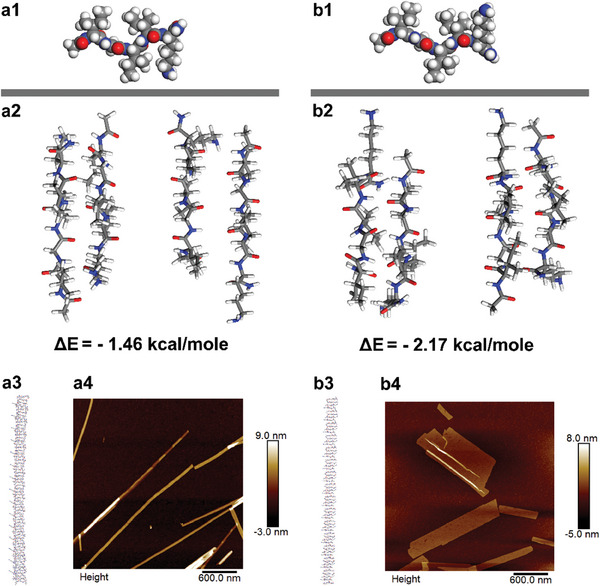
Simulation and experimental results for a1–a4) *^Da^* IG*^L^* I_2_ *^L^* K and b1–b4) *^Da^* IG*^L^* I_2_ *^D^* K. a1,b1) Stable molecular conformations. a2,b2) Stable dimers. a3,b3) Multi‐stranded *β*‐sheets. a4,b4) Peptides nanostructures.

For *^L^* IG*^L^* I_2_ *^L^* K and *^L^* IG*^L^* I_2_ *^D^* K, their N‐terminal backbone bending was mainly driven by the interactions between their N‐terminal backbone and the Ile4 side chain. Hence, the Ile1 side chain at the N‐terminal appeared to be excluded from these bent structures (Figures 2i,j and 3). Thus, we assumed that substitution for this L‐form Ile1 with D‐allo‐form Ile (denoted as *^Da^* I or *^Da^* Ile) likely led to destabilization of the N‐terminal backbone bending due to the steric hindrance of the *^Da^* Ile1 side chain. AFM measurements indicated *^Da^* IG*^L^* I_2_ *^L^* K formed nanoribbons with a relatively uniform width of ≈70 nm and a height of 6 nm while *^Da^* IG*^L^* I_2_ *^D^* K formed giant nanosheets with widths as wide as several hundreds of nanometers and heights of ≈3 nm (**Figure** **6**a4,b4; Figure S5, Supporting Information). Importantly, these flat nanostructures did not show any helical handedness.

Simulations of single *^Da^* IG*^L^* I_2_ *^L^* K and *^Da^* IG*^L^* I_2_ *^D^* K molecules did reveal loss of the N‐terminal backbone bending, and instead, their stable molecular conformations were extended (Figure 6a1,b1). Upon dimerization, it was interesting that two stable dimers were observed for *^Da^* IG*^L^* I_2_ *^L^* K or *^Da^* IG*^L^* I_2_ *^D^* K, with the Lys5 and *^Da^* Ile1 residues shifting out, respectively (Figure 6a2,b2). Furthermore, the two dimers showed a small binding energy gap, −1.46 and −2.17 kcal mol^−1^ for *^Da^* IG*^L^* I_2_ *^L^* K and *^Da^* IG*^L^* I_2_ *^D^* K, respectively. Further stacking along the interstrand H‐bonding direction led to right‐ and left‐handed *β*‐sheets, for the dimers with the Lys5 and *^Da^* Ile1 residue shifting out, respectively (Figure S6, Supporting Information), in conflict with our experimental observations. Due to the small energy difference between the two dimers of *^Da^* IG*^L^* I_2_ *^L^* K or *^Da^* IG*^L^* I_2_ *^D^* K, we then constructed mixed sheets using the Kabsch algorithm, with the two dimers packing alternately. After structural optimization, the mixed *β*‐sheets displayed flat morphologies (Figure 6a3,b3), presumably due to the shape complementarity of the two dimers with opposite deflection. Subsequent lamination of these flat *β*‐sheets should eventually result in the formation of wide ribbons with suprastructural chirality racemization, highly consistent with the experimental results (Figure 6a4,b4).

Additionally, given the vital role of Gly in the backbone bending of *^L^* IG*^L^* I_2_ *^L^* K and *^L^* IG*^L^* I_2_ *^D^* K, we synthesized *^L^* I*^L^* A*^L^* I_2_ *^L^* K and *^L^* I*^L^* A*^L^* I_2_ *^D^* K by substituting alanine (Ala or A) for Gly. Due to the lack of the highly flexible Gly, the two single peptides adopted an extended conformation, and Lys5 shifting out was observed upon dimerization in our simulations (Figure S7a,b,e,f, Supporting Information). Further stacking of the dimers along the interstrand H‐bonding direction led to left‐handed *β*‐sheets (Figure S7c,g, Supporting Information). Consistent with these simulated structures, AFM imaging revealed left‐handed nanofibrils formed by the two peptides (Figure S7d,h, Supporting Information).

## 3 Conclusion

Given the multiple roles of flexible and achiral Gly in protein architecture and function, we here designed a group of pentapeptides with different Gly insertions and chiral substitutions in a self‐assembling sequence I_3_K, and investigated their impacts on molecular conformations, *β*‐sheet assembly, and final nanostructures and suprastructural handedness. We demonstrated that different bent and extended structures could be generated by Gly insertion and the bending degree was related to the insertion position. When the Gly residue was located at the middle of the pentapeptide sequence, the resulting peptides *^L^* I_2_G*^L^* I*^L^* K and *^L^* I_2_G*^L^* I*^D^* K tended to take extended conformations. The rest peptides with the Gly residue away from the middle position adopted both bent and extended structures, with the dominant one being determined by their total interaction energies. Both *^L^* IG*^L^* I_2_ *^L^* K and *^L^* IG*^L^* I_2_ *^D^* K showed significant bending, driven by stronger vdW interactions between the N‐terminal backbone and the Ile4 side chain. Distinct from the widely studied *β*‐turns or loops driven by intrastrand H‐bonding, the bent structures in these short Gly‐containing pentapeptides were primarily stabilized by vdW interactions between either the side chains themselves or the side chain and the local backbone around the inserted Gly.

When the bent and extended monomers packed along the H‐bonding direction to form dimers and multistranded *β*‐sheets, different interstrand H‐bonding modes and residue registry shifts were obtained. For the *β*‐sheets with the C‐terminal Lys5 residues shifting out without the constraint of interstrand H‐bonding, the twisting direction of these sheets and the final nanofibrils (*β*‐sheet lamination) was dictated by the rest residues, irrespective of the chirality of Lys5 (chiral truncation). For the *β*‐sheets with the Ile1 residues shifting out, the Lys5 residues at the C‐termini did participate in interstrand H‐bonding and the twisting direction of these sheets and nanofibrils was controlled by the chirality of the C‐terminal Lys.

The chiral substitution for the L‐form Ile1 of *^L^* IG*^L^* I_2_ *^L^* K or *^L^* IG*^L^* I_2_ *^D^* K with D‐allo‐form Ile caused a conformational transition from bent to extended structures, by altering the Ile side chain orientation and imposing steric hindrance on the N‐terminal backbone bending. Upon dimerization, two stable extended dimers with the Lys5 and *^Da^* Ile1 residue shifting out, respectively, were obtained. Because of their similar energies, the two distinct dimers were proposed to pack alternately, leading to flat *β*‐sheets and final wide ribbons with suprastructural chirality racemization.

Overall, this study not only helps understand the specific roles of the flexible Gly in protein architecture, in particular, in structural diversity and polymorphism complexity, but also serves as a paradigm for tuning molecular conformations and supramolecular peptide architectures and handedness via shifting the Gly position within the sequence.

## 4 Experimental Section

### Materials and Sample Preparation

All the peptides used were obtained from China Peptides Co., Ltd. (www.chinapeptides.com), with a purity of >98%. Note that in order to avoid the electrostatic effect of the terminal carboxylate and amine groups, these peptides were capped through amidation at their C‐termini and acetylation at their N‐termini during synthesis. These peptides showed excellent solubility upon dissolution in water at concentrations of <32 mm and pH of 7.0. Prior to AFM and TEM characterizations, the peptide solutions were incubated at least for one week at room temperature.

### AFM and TEM Characterizations

AFM measurements were performed using a Bruker MultiMode 8 scanning probe microscope with a NanoScope V controller at room temperature. Prior to AFM imaging, samples were deposited on a freshly cleaved mica substrate. Height, amplitude, and phase images were obtained simultaneously in ScanAsyst mode in air and presented after the first‐order flattening. TEM measurements were carried out using a JEOL‐1400 electron microscope with an accelerating voltage of 120 kV. For TEM imaging, samples were placed on a copper grid coated with a carbon support film and stained with 2% uranyl acetate.

### Simulations

For each peptide, various random molecular conformations were first generated using LAMMPS (see Supporting Information for the details of the MD simulation).^[^ ^19^ ^]^ In light of the chain bending or extending degree and the corresponding energy calculation, which were obtained using the Molclus programme,^[^ ^16^ ^]^ 50 stable strand conformations were then selected for further simulations. Single strand conformations with the lowest energies were obtained after DFT structural optimization and energy calculation.

Density functional theory (DFT) simulations were conducted using Gaussian 09 software,^[^ ^20^ ^]^ with the generalized gradient approximation (GGA) scheme and the PBE0 exchange−correlation function.^[^ ^21^ ^]^ The molecular structure was optimized by the def2‐SVP basis set (2‐ζ base group), and conformational energy was calculated by the def2‐ TZVP basis set (3‐ζ base group).^[^ ^22^ ^]^ During the whole DFT simulation process, the DFT‐D3 dispersion correction was employed.^[^ ^23^ ^]^

Based on the stable strands, 500 antiparallel dimers were generated by Molclus. These dimers were structurally optimized using a semiempirical QC method, GFN2‐xTB,^[^ ^24^ ^]^ and 10 dimeric configurations with lower energies were selected for further DFT optimization, giving rise to dimer structures with the lowest energies. In the structural optimization of dimers, the counterpoise method developed by Boys and Bernardi, was applied to accomplish the Basis Set Superposition Error (BSSE) correction.^[^ ^25^ ^]^

Based on the stable dimers, multi‐stranded *β*‐sheets were constructed using the Kabsch algorithm,^[^ ^18^ ^]^ followed by the optimization of their twisting via the GFN2‐xTB method. Note that in our simulations, VMD software was utilized to visualize all the snapshots,^[^ ^26^ ^]^ and the electronic wavefunction analysis was performed by Multiwfn.^[^ ^27^ ^]^

## Conflict of Interest

The authors declare no conflict of interest.

## Supporting information



Supporting Information

## Data Availability

The data that support the findings of this study are available from the corresponding author upon reasonable request.
