# Supplementary material for: How Do Glycine‐Induced Bent Structures Influence Hierarchical Nanostructuring and Suprastructural Handedness in Short Peptide Assembly?
Source: Adv Sci (Weinh). 2025 Feb 25;12(15):2413602. doi: 10.1002/advs.202413602 (PMC12005805; doi:10.1002/advs.202413602)
Supplement: Supplementary file 1 — Supporting Information [file ADVS-12-2413602-s001.docx]

*Supporting Information*

How do Glycine-Induced Bent Structures Influence Hierarchical Nanostructuring and Suprastructural Handedness in Short Peptide Assembly?

*Xinfeng Ju, Kai Qi, Yan Wang, Limin Zhang, Yingyu Wang, Muhan Wang^*^, Jiqian Wang, Jun Zhang, Jian R. Lu, Hai Xu^*^, and Yurong Zhao^*^*

Corresponding Author E-Mails: [wangmuhan@qut.edu.cn](mailto:wangmuhan@qut.edu.cn) (M.W.)

xuh@upc.edu.cn (H.X.)

yurongzhao@upc.edu.cn (Y.Z.)

1. **MD simulations**

We used molecular dynamics (MD) simulations to generate a batch of molecular conformations for each peptide. For the initial configuration, there was a single peptide molecule and 4000 water molecules in a 48 Å × 48 Å × 48 Å water-filled box. The large-scale atomic/molecular massively parallel simulator (LAMMPS) software was used to perform MD simulations.^[1]^ Inter- and intra-atomic interactions were described by the all-atom (AA) force field of the optimized potentials for liquid simulations (OPLS-AA) force field, which were composed of pairwise and bonding interactions.^[2]^ The SPC/E model was employed to describe water molecules.^[3]^ The OPLS-AA force field ss expressed as:

| $E_{\mathrm{total}}= E_{\mathrm{bonds}}+ E_{\mathrm{angles}}+ E_{\mathrm{dihedrals}}+E_{\mathrm{nonbonded}}$ | (1) |
| --- | --- |
| $E_{\mathrm{bonds}}= \sum_{\mathrm{bonds}} K_{r}{(r-r_{0})}^{2}$ | (2) |
| $E_{\mathrm{angles}}=\sum_{\mathrm{angles}} K_{\theta}{(\theta-\theta_{0})}^{2}$ | (3) |
| $E_{\mathrm{dihedrals}}=\sum_{\mathrm{dihedrals}} (\frac{V_{1}}{2} \left[ 1+\cos\left( \varphi-\varphi_{1} \right) \right]+\frac{V_{2}}{2} \left[ 1-\cos\left( 2\varphi-\varphi_{2} \right) \right]+ \frac{V_{3}}{2} \left[ 1+\cos\left( 3\varphi-\varphi_{3} \right) \right]+\frac{V_{4}}{2} \left[ 1-\cos\left( 4\varphi-\varphi_{4} \right) \right])$ | (4) |
| $E_{\mathrm{nonbonded}}=\sum_{i>j} \left[ \varepsilon_{ij}\left( \frac{\sigma^{12}}{r_{ij}^{12}}- \frac{\sigma^{6}}{r_{ij}^{6}} \right)+ \frac{{q_{i}q_{j}e}^{2}}{4\pi\varepsilon_{0}r_{ij}} \right]$ | (5) |

*E*_total_ is the total energy in the system, which is equal to the energy of bond stretching (*E*_bonds_) plus angles shake (*E*_angles_) plus dihedrals shake (*E*_dihedrals_) plus pairwise (*E*_nonbonded_).

The whole MD simulation process included two steps. First, 1 ns NPT ensemble was performed at 298 K and 0.1 MPa to obtain a reliable system density. Second, 2 ns simulations in NVT ensemble were performed for sampling various peptide conformations. The temperature and the pressure were controlled by the Nosé thermostat and barostat, respectively.^[4]^ The long-range electrostatic interactions were treated using the PPPM summation.^[5]^ The time step was set as 1 fs. Full trajectories were saved and the frames were output every 1 ps for sampling the results. All the snapshots were displayed using the VMD software.^[6]^

1. **EDA-FF**

Energy decomposition analysis based on forcefield (EDA-FF) is a method that can provide detailed information about interatomic or interfragment interactions, on the basis of the molecular forcefield. Specifically, the parameters in AMBER forcefield were employed in our EDA-FF calculations. The non-bonded interaction is resolved into electrostatic and van der Waals (vdW) interactions. The electrostatic interaction (ele) is described by Coulomb’s law. The vdW interaction is evaluated using common Lennard-Jones 12-6 potential, and is divided into exchange repulsion and attractive dispersion. The AMBER forcefield that was used to calculate the electrostatic (ele), exchange repulsion (rep), and dispersion (disp) components of non-bonded interactions between atoms is described by the following formula:

$$E_{\mathrm{AB}}^{\mathrm{ele}}=\frac{q_{A}q_{B}}{r_{\mathrm{AB}}}$$

$$E_{\mathrm{AB}}^{\mathrm{vdW}}=\varepsilon_{\mathrm{AB}}\left( \frac{R_{\mathrm{AB}}^{0}}{r_{\mathrm{AB}}} \right)^{12}-2\varepsilon_{\mathrm{AB}}\left( \frac{R_{\mathrm{AB}}^{0}}{r_{\mathrm{AB}}} \right)^{6}$$

$$E_{\mathrm{AB}}^{\mathrm{rep}}=\varepsilon_{\mathrm{AB}}\left( \frac{R_{\mathrm{AB}}^{0}}{r_{\mathrm{AB}}} \right)^{12}$$

$$E_{\mathrm{AB}}^{\mathrm{disp}}=-2\varepsilon_{\mathrm{AB}}\left( \frac{R_{\mathrm{AB}}^{0}}{r_{\mathrm{AB}}} \right)^{6}$$

where A, B is the atomic label, *q* is the atomic charge, *r* is the interatomic distance, *ε* is the vdW potential well depth, *R*^0^ is the non-bonded distance between atoms, and when *r* = *R*^0^, the vdW energy between atoms is exactly equal to the potential well depth.

1. **Supplementary Figures**


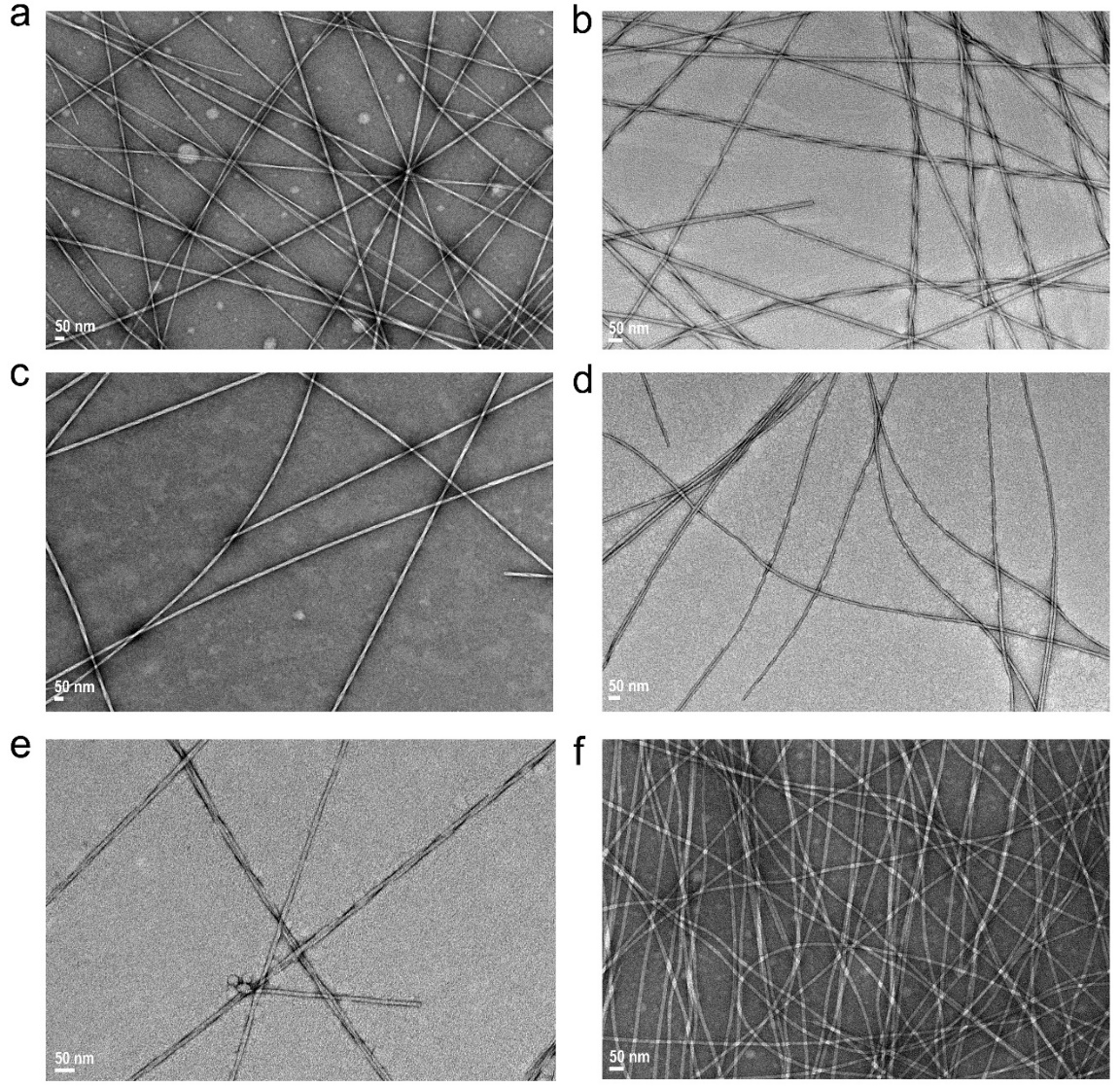


**Figure S1.** Negative-staining TEM images of nanofibrils self-assembled from a) *^L^*I_3_G*^L^*K, b) *^L^*I_3_G*^D^*K, c) *^L^*I_2_G*^L^*I*^L^*K, d) *^L^*I_2_G*^L^*I*^D^*K, e) *^L^*IG*^L^*I_2_*^L^*K, and f) *^L^*IG*^L^*I_2_*^D^*K. The peptide solutions (8 mM and pH of 7.0) were incubated for 1 week at ambient conditions prior to TEM measurements. Notice that although these peptide nanofibrils all showed clear helical handedness in their AFM images (Figure 1 of the main text), it was difficult to discern the twisting by TEM for some nanofibrils, possibly due to the effect of staining, as well as their different twisting degrees.


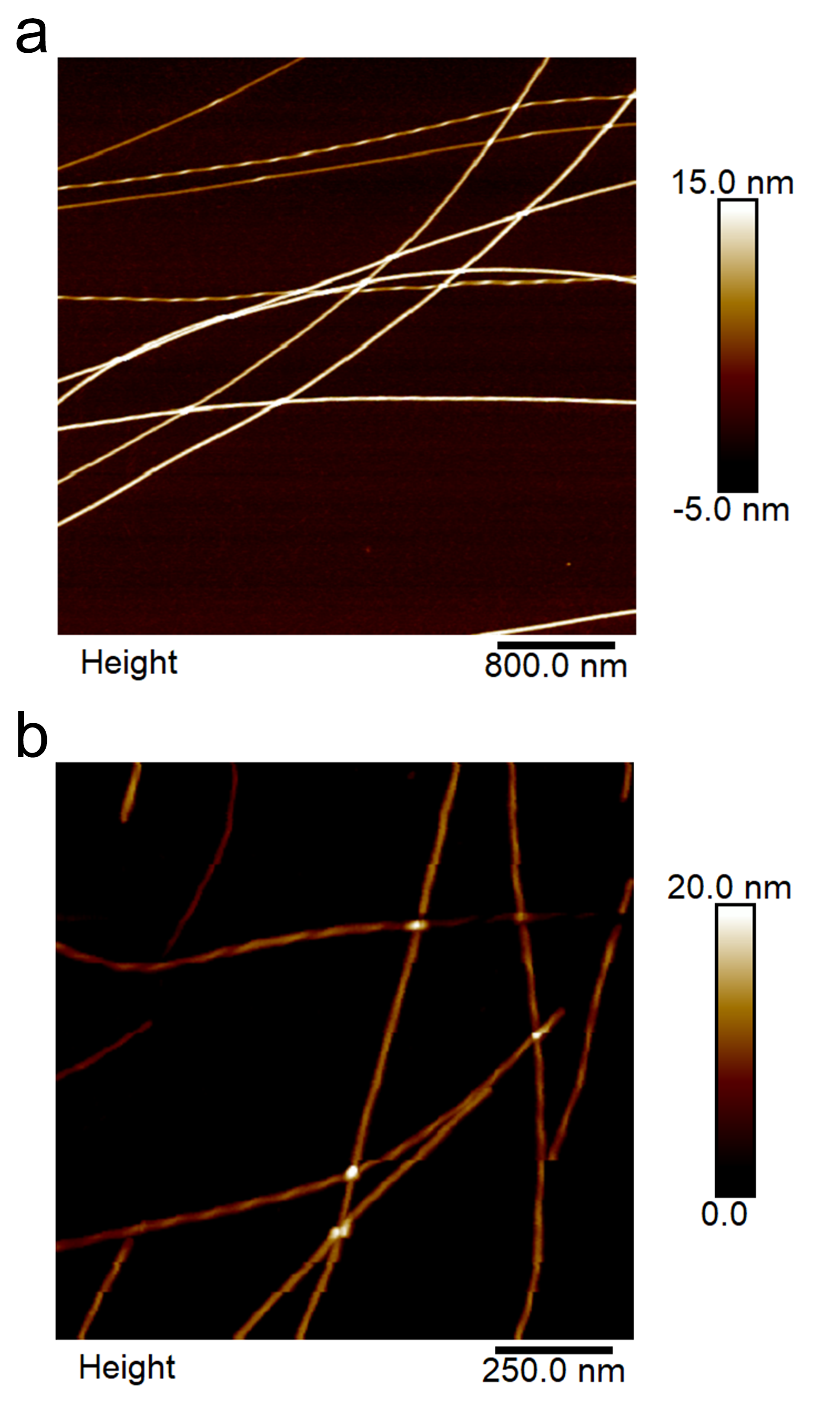


**Figure S2.** Left-handed and flat nanofibrils formed by a) *^L^*I_3_G*^L^*K and b) *^L^*I_3_G*^D^*K.


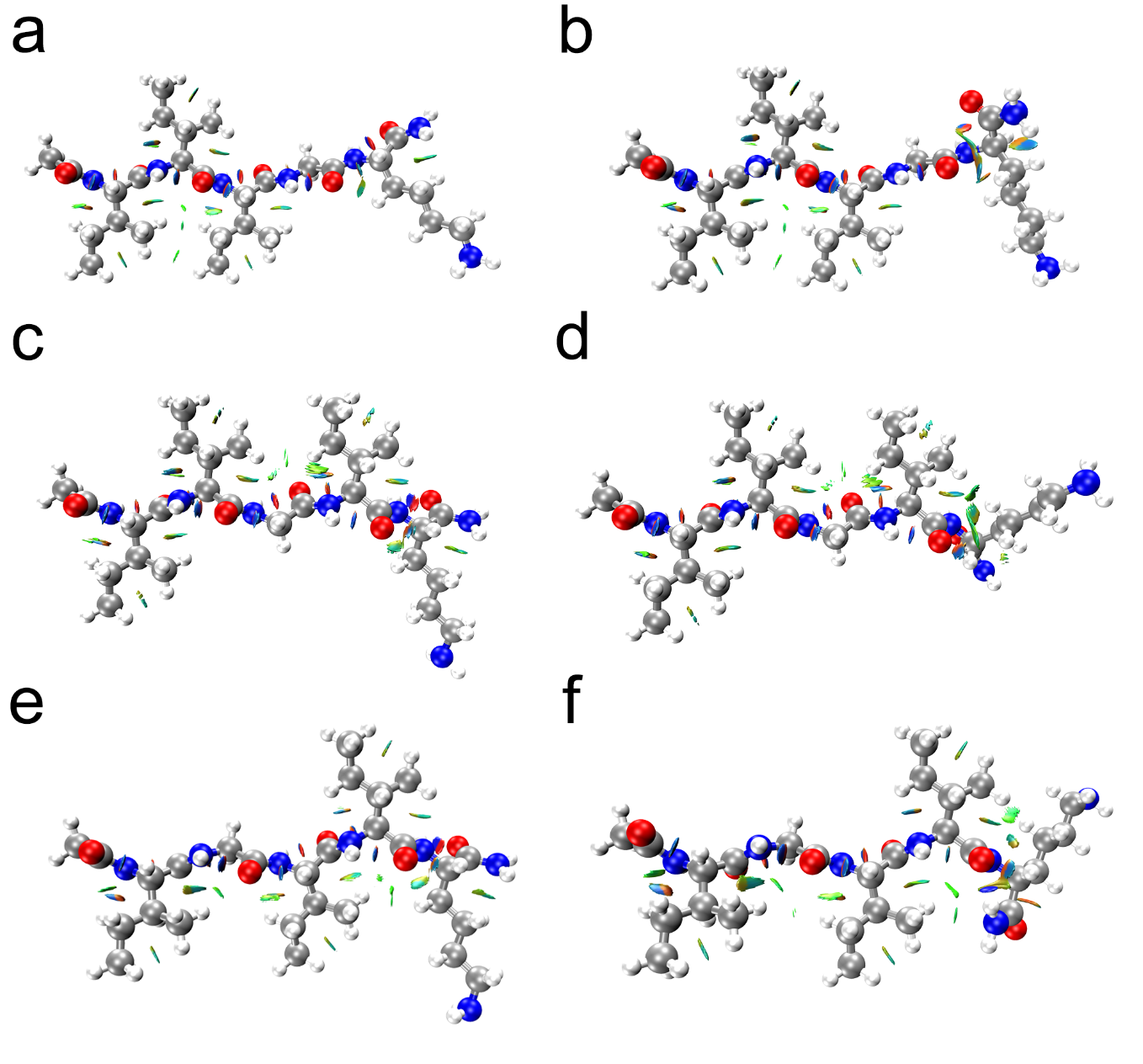


**Figure S3.** RDG isosurfaces for the extended chains of a) *^L^*I_3_G*^L^*K, b) *^L^*I_3_G*^D^*K, c) *^L^*I_2_G*^L^*I*^L^*K, d) *^L^*I_2_G*^L^*I*^D^*K, e) *^L^*IG*^L^*I_2_*^L^*K, and f) *^L^*IG*^L^*I_2_*^D^*K. Atoms coloring scheme for molecular structures is: red, oxygen; blue, nitrogen; white, hydrogen, and gray, carbon. Red, green and blue patches in the RGD isosurfaces represent repulsive steric hindrance, vdW interactions, and attractive H-bonding, respectively.


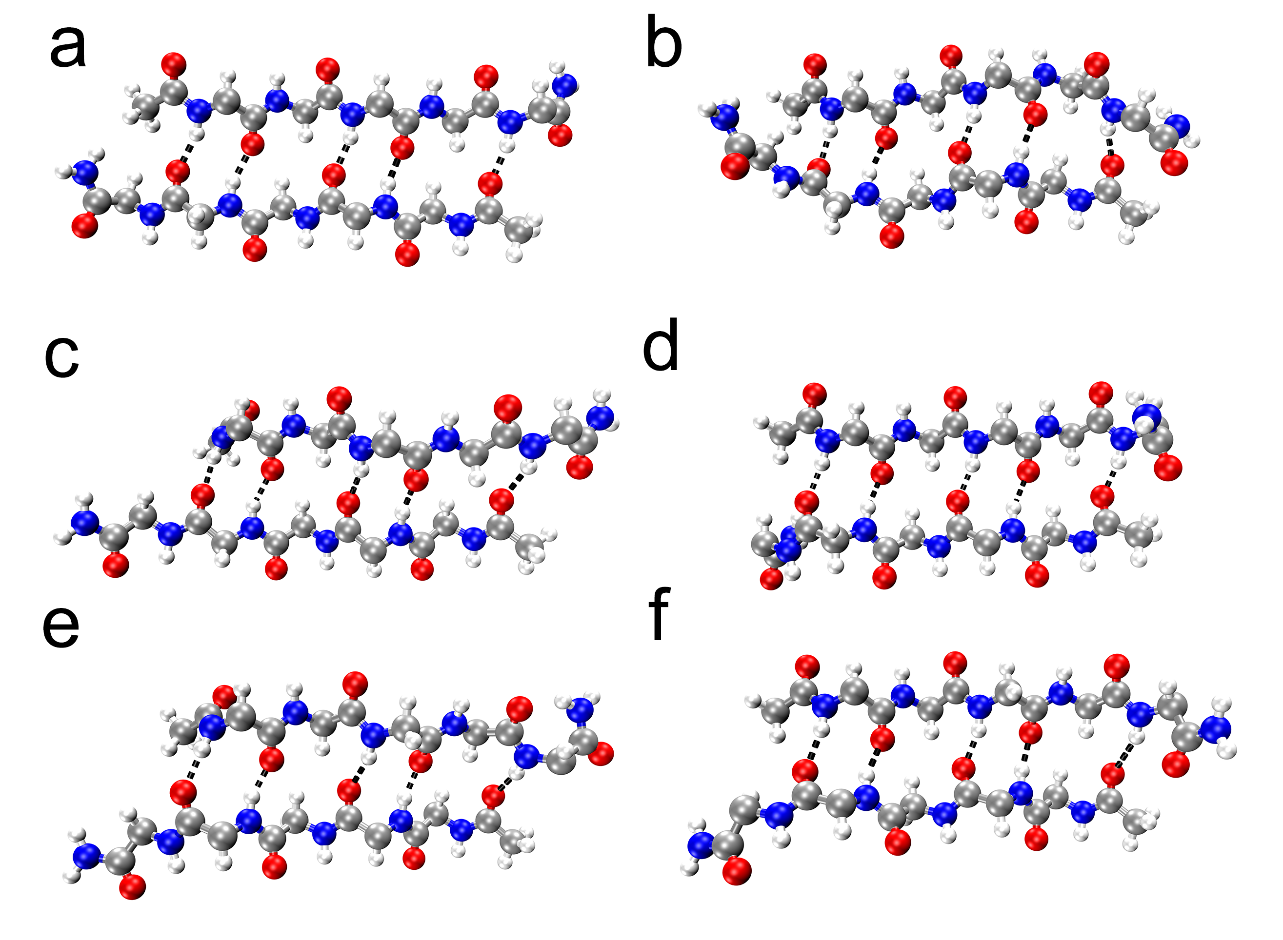


**Figure S4.** a,b) Lys5 shifting out and interstrand H-bonding within the extended and bent dimers of *^L^*I_3_G*^L^*K, respectively. c,d) Lys5 shifting out and interstrand H-bonding within the extended and bent dimers of *^L^*I_3_G*^D^*K, respectively. e,f) Lys5 shifting out and interstrand H-bonding within the *^L^*I_2_G*^L^*I*^L^*K and *^L^*I_2_G*^L^*I*^D^*K extended dimers, respectively


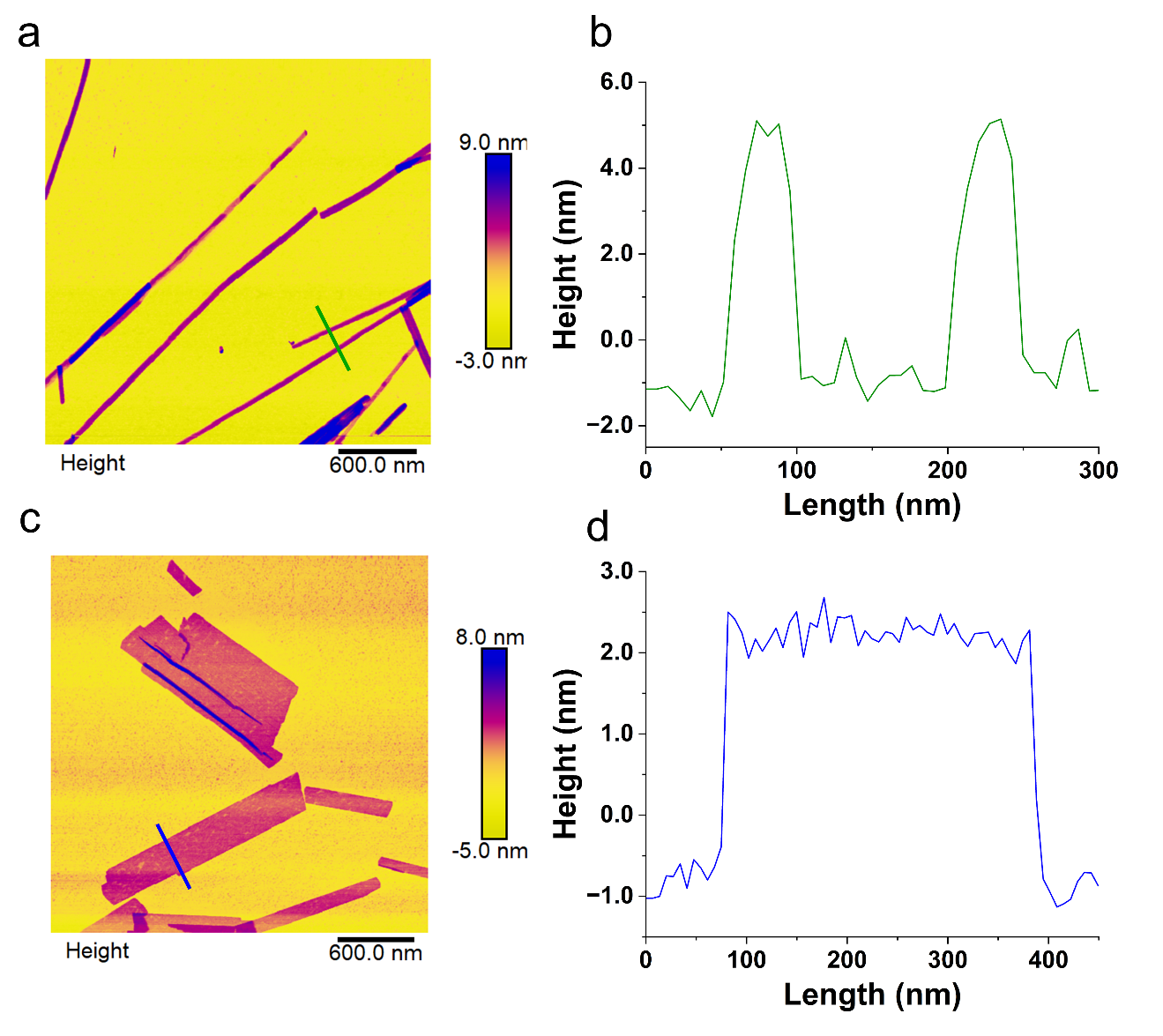


**Figure S5.** a) AFM height image of *^Da^*IG*^L^*I_2_*^L^*K nanoribbons and b) the representative height profile along the green line indicated in (a). c) AFM height image of *^Da^*IG*^L^*I_2_*^D^*K nanosheets and d) the representative height profile along the blue line indicated in (c). Such a height profiling was performed by using the NanoScope Analysis 1.80 software (Bruker).


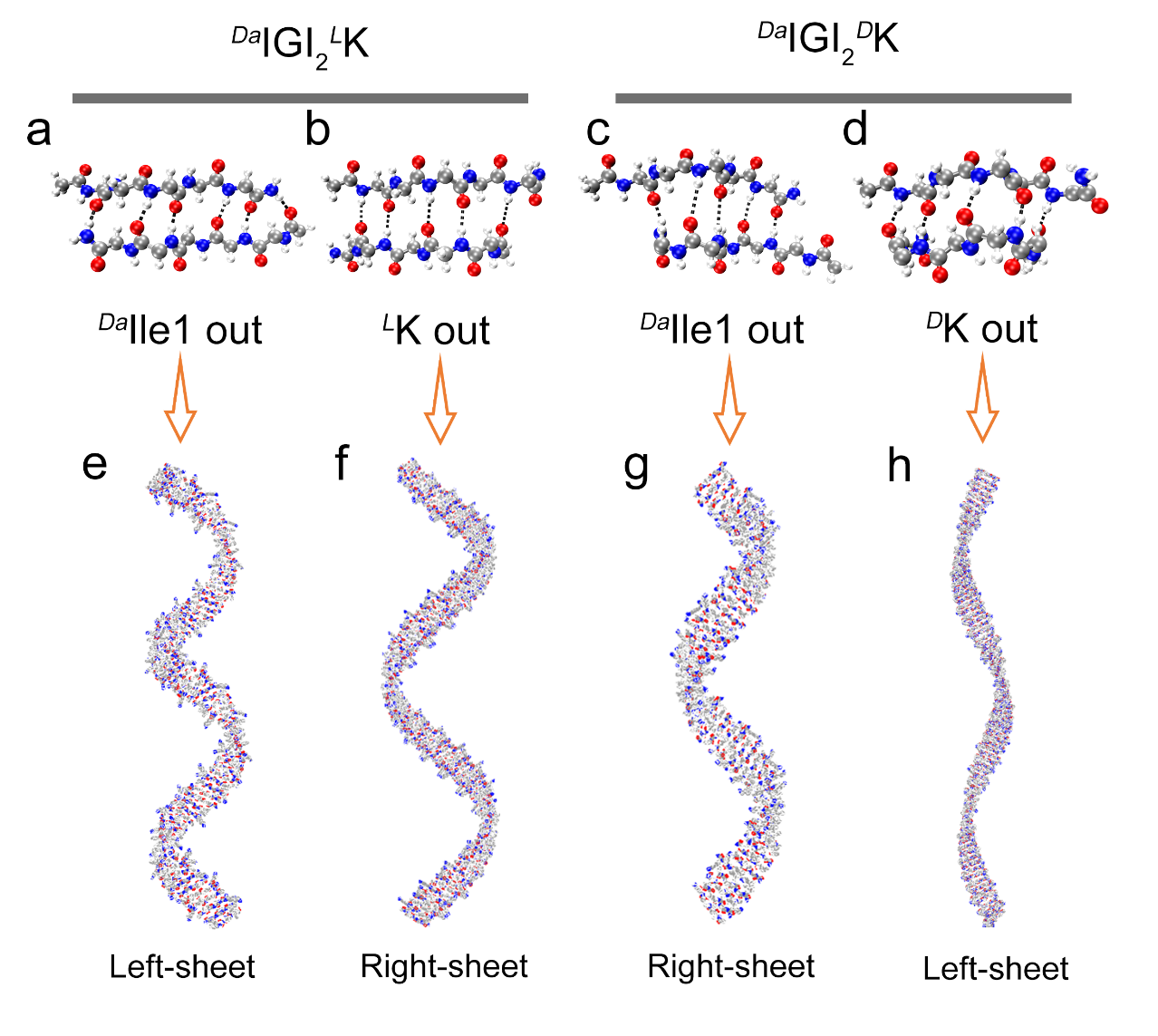


**Figure S6.** a,b) Two stable dimers of *^Da^*IG*^L^*I_2_*^L^*K, with the *^Da^*Ile1 and Lys5 residues shifting out, respectively and multi-strand *β*-sheets. c,d) Two stable dimers of *^Da^*IG*^L^*I_2_*^D^*K, with the *^Da^*Ile1 and Lys5 residues shifting out, respectively and multi-strand *β*-sheets. e-h) Multi-stranded *β*-sheets evolved from the dimers showed in (a-d), respectively.


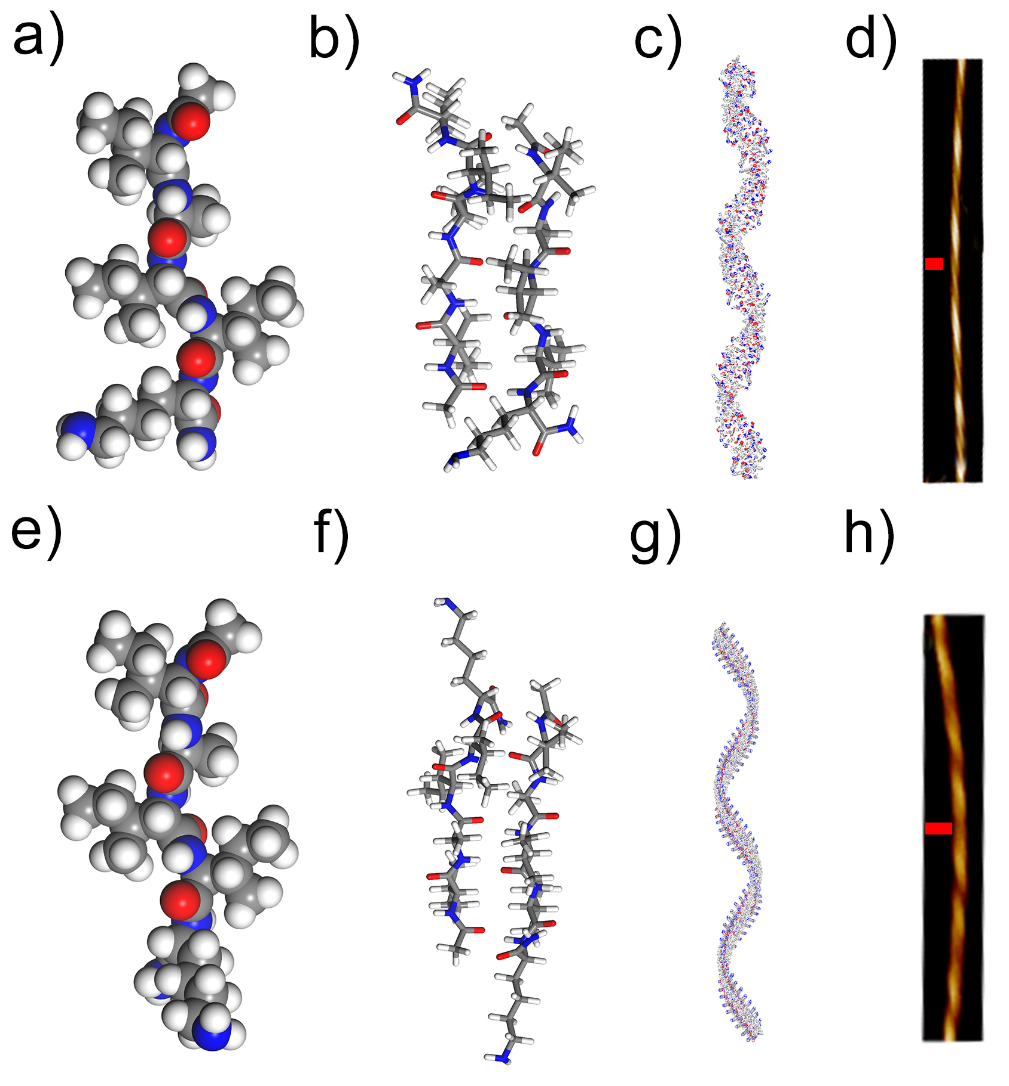


**Figure S7.** Simulation and experimental results for a-d) *^L^*I*^L^*A*^L^*I_2_*^L^*K and e-h) *^L^*I*^L^*A*^L^*I_2_*^D^*K. a,e) Stable monomer conformations. b,f) Stable dimers. c,g) Multi-stranded *β*-sheets. d,h) Fibrils and their suprastructural handedness determined from AFM measurements. Red scale bar: 50 nm.

1. **References**
2. S. Plimpton, *J. Comput. Phys.* **1995**, *117*, 1-19.
3. a) W. L. Jorgensen, J. Tirado-Rives, *J. Am. Chem. Soc.* **1988**, *110*, 1657-1666; b) W. L. Jorgensen, D. S. Maxwell, J. Tirado-Rives, *J. Am. Chem. Soc.* **1996**, *118*, 11225-11236.
4. H. J. Berendsen, J.-R. Grigera, T. P. Straatsma, *J. Phys. Chem.* **1987**, *91*, 6269-6271.
5. a) S. Nosé, *J. Chem. Phys.* **1984**, *81*, 511-519; b) G. J. Martyna, D. J. Tobias, M. L. Klein, *J. Chem. Phys.* **1994**, *101*, 4177-4189.
6. R. W. Hockney, S. Goel, J. Eastwood, *J. Comput. Phys.* **1974**, *14*, 148-158.
7. W. Humphrey, A. Dalke, K. Schulten, *J. Mol. Graphics* **1996**, *14*, 33-38.
